# Anterior Segment Optical Coherence Tomography and its Clinical Applications in Glaucoma

**DOI:** 10.5005/jp-journals-10008-1109

**Published:** 2012-08-16

**Authors:** Haitao Li, Vishal Jhanji, Syril Dorairaj, Andrea Liu, Dennis SC Lam, Christopher K Leung

**Affiliations:** 1 Professor, Department of Ophthalmology and Visual Sciences, Hong Kong Eye Hospital, The Chinese University of Hong Kong, Hong Kong; 2 Professor, Department of Ophthalmology and Visual Sciences, Hong Kong Eye Hospital, The Chinese University of Hong Kong, Hong Kong; 3 Professor, Department of Ophthalmology, Mayo Clinic, Jacksonville, Florida, USA; 4 Professor, Department of Ophthalmology and Visual Sciences, Hong Kong Eye Hospital, The Chinese University of Hong Kong, Hong Kong; 5 Professor, Department of Ophthalmology and Visual Sciences, Hong Kong Eye Hospital, The Chinese University of Hong Kong, Hong Kong; 6 Professor, Department of Ophthalmology and Visual Sciences, Hong Kong Eye Hospital, The Chinese University of Hong Kong, Hong Kong

**Keywords:** Optical coherence tomography, Anterior segment, Anterior chamber angle.

## Abstract

Anterior segment optical coherence tomography (ASOCT) is an imaging modality that provides both quantitative and qualitative information on the cornea, anterior chamber angle, iris and crystalline lens. ASOCT shows excellent repeatability and reproducibility for the measurement of corneal thickness, anterior chamber angle and anterior chamber depth. With realtime video recording, ASOCT can also effectively capture the dynamic changes of the iris and lens in response to light and eye accommodation. With this additional information, physiological and pathological changes of the anterior segment structures are now better understood. This article aims to provide an overview of the current applications of ASOCT in glaucoma.

**How to cite this article:**

Li H, Jhanji V, Dorairaj S, Liu A, Lam DSC, Leung CK. Anterior Segment Optical Coherence Tomography and its Clinical Applications in Glaucoma. J Current Glau Prac 2012;6(2):68-74.

## INTRODUCTION

Assessment of the anterior segment is an integral part of ophthalmic examination. Traditionally, the anterior segment and iridocorneal angle are evaluated with the help of a slit lamp and gonioscope. With the advancement of technology, low coherence interferometry was developed, upon which optical coherence tomography (OCT) is based. OCT provides multiple detailed cross-sectional images of the internal structures in biological tissues. Currently, two anterior segment OCT (AS-OCT) models are commercially available-the Visante OCT (Carl Zeiss Meditec, Dublin CA, USA) and the slit-lamp OCT (SLOCT) (Heidelberg Engineering, GmbH, Dossenheim, Germany). Since their availability, noninvasive studies of the structures and pathologies of the anterior segment have been made possible. This article serves to highlight the principles and clinical applications of ASOCT in glaucoma.

## PRINCIPLES OF AS-OCT

The commercially available OCT system, also known as time-domain OCT (TD-OCT), was first described in 1991 by Huang et al.^1^ The principle of OCT imaging is based on the measurement of delay in light reflected from various tissue structures. Low coherence infrared light is split by the beam splitter of a Michelson interferometer into two components―one is directed to a movable mirror in the reference arm and the other to the object of interest in the sample arm. The reflected signals from these two components are then superimposed at the interferometer. The strength of each reflected signal is a function of depth in each scan.

Ophthalmic OCT was initially developed for retinal imaging and used a near-infrared 800 nm superluminescent diode (SLD) as the light emitting source. This OCT model was subsequently applied to anterior segment imaging, capturing images of the cornea, anterior chamber angle, iris and crystalline lens.^2-7^ However, this type of OCT was limited by its light source and imaging speed (400 A-scans per second). This shortcoming prevents the light beam from penetrating the limbus and other opaque structures.^3^ To overcome this drawback, AS-OCT, specially designed for anterior segment imaging was developed. This new model employs an SLD of a longer wavelength (1310 nm). With the use of this model, about 90% of the 1310 nm light is absorbed by the ocular media before hitting the retina, allowing us to use light that is 20 times stronger before reaching the retinal exposure limit. With the improvements in speed and light penetration, the entire anterior segment can now be captured in one frame, in 0.125 seconds and in 18 μm resolution (Visante™ OCT User Manual, 2006). However, the ciliary body is still poorly visualized owing to the pigmented posterior layer of the iris which hinders light penetration.^8,9^

Inspite of all the above-mentioned advantages, TD-OCT is relatively time-consuming mainly due to its sliding a mirror along the reference arm. Currently, the fastest commercial TD-OCT system is the Visante anterior segment OCT (Carl Zeiss Meditec, Inc, Dublin, CA), which acquires 2000 A-scans per second. With the introduction of the Fourier domain OCT (FD-OCT), ocular imaging can be performed at a higher speed and at a higher resolution.^10-12^ The stated axial resolution of FD-OCT ranges from 4 to 7 μm. Some of the food and drug administration (FDA)-approved commercially available spectral domain OCT machines became available at the end of year 2006 and include the following: RTVue (Optovue Inc, Fremont, California), Cirrus HD-OCT (Carl Zeiss Meditec Inc, Dublin, California), Spectralis (Heidelberg Engineering Inc, Heidelberg, Germany), SOCT Copernicus (Optopol Technology, Zawiercie, Poland) and 3D OCT-1000 (Topcon, Paramus, New Jersey).

## AS-OCT MODELS

At present, two AS-OCT models are commercially available (Table 1)―the ZEISS Visante™ OCT Model 1000 (Visante OCT) (Carl Zeiss Meditec, Dublin CA, USA) and the Slit-Lamp OCT (SLOCT) (Heidelberg Engineering, GmbH, Dossenheim, Germany). Both equipped with a 1310 nm SLD, they provide rapid, noninvasive imaging of the anterior segment with the patient in upright position.

### ZEISS Visante™ OCT Model 1000 (Visante OCT)

The Visante OCT is a standalone machine and was approved by the FDA in September 2005. Its scan rate reaches up to 2048 A-scans per second and its axial and transverse image resolutions are up to 18 and 60 μm. (Visante™ OCT User Manual, 2006). The Visante OCT can be easily operated and does not require much experience for image acquisition.

The Visante ASOCT machine can be operated in various modes, each of which is configured for a specific application, e.g. cornea, anterior chamber biometry. To further its technological superiority, calculations of the depth and width of anterior chamber and anterior chamber angle (ACA) can all be done automatically or manually by the caliper tools provided.

### Slit-lamp OCT (SLOCT)

The SLOCT is essentially an OCT incorporated with Haag-Streit slit-lamp biomicroscopy (SLOCT User Manual, 2006). Its axial and transverse resolutions are 25 μm and 20 to 100 μm, respectively (SLOCT Operation Manual, 2006). Parameters including the central corneal thickness (CCT), the central anterior chamber depth (ACD) and the anterior chamber volume (ACV) can all be determined automatically. After manual location of the scleral spur, the interscleral spur distance, angle opening distance (AOD 500, AOD 750), trabecular-iris spur area (TISA 500, TISA 750), trabecular-iris angle (TIA 750) and the scleral spur-to-scleral spur distance are measured semiautomatically.

## CLINICAL APPLICATIONS OF ASOCT

### Imaging of Anterior Chamber

Anterior Chamber angle (ACA)

**Fig. 1:**
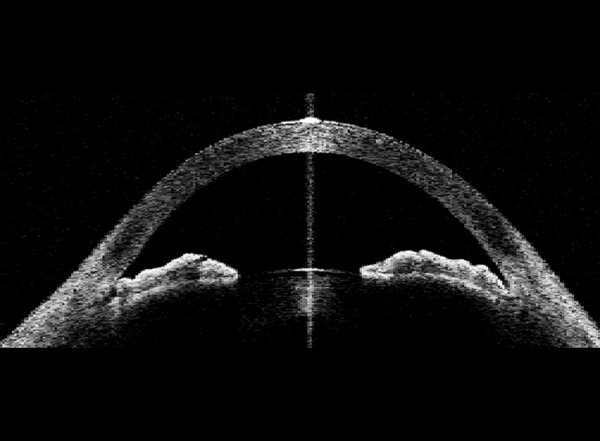
Iris configuration in narrow angles: Convex configuration or anterior bowing of iris is visualized in one frame

**Figs 2A and B:**
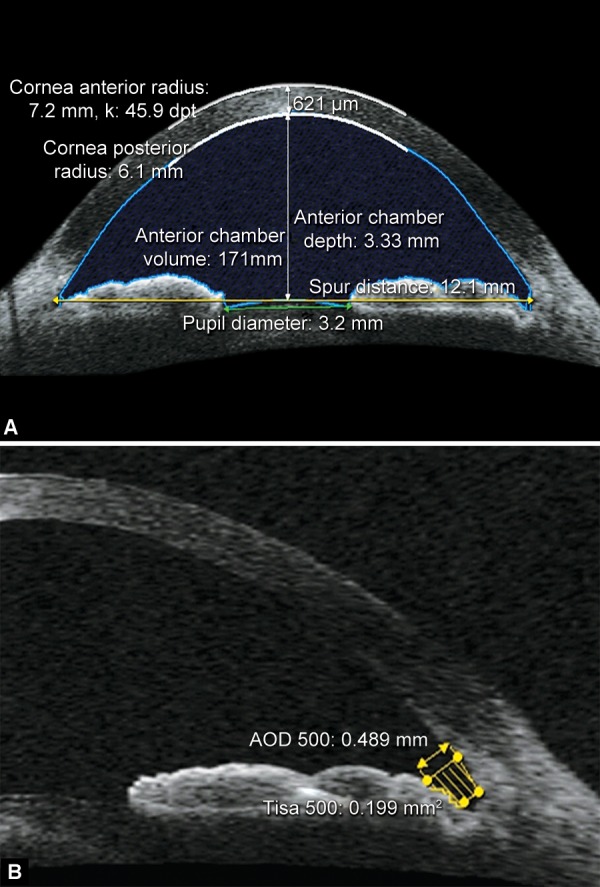
(A) Measurement of chamber parameters using AS-OCT: Anterior chamber depth and volume can be measured using AS-OCT. (B) AOD500 (angle opening distance at 500 μm from the scleral spur), TISA500 (trabecular-iris space area at 500 μm from the scleral spur), and TIA500 (trabecular-iris angle at 500 μm from the scleral spur) can be measured using AS-OCT

Primary angle closure is the leading cause of glaucoma-induced blindness in the East Asian population.^13^ Evaluation of the width of the ACA and its inlet during the ophthalmic examination is essential to determining the susceptibility of the angle to close (Fig. 1). Although gonioscopy is the current reference standard in ACA assessment, its drawbacks include its subjective and semiquantitative results, its need for illumination, its dependence on the operator and its inability to delineate the angle configuration when the goniolens is in direct contact with the cornea.^14,15^ Quantitative assessment of the ACA was first described by Pavlin and Foster in ultrsound biomicroscopy (UBM) imaging.^16^ The most important parameters are the angle opening distance (AOD) and the trabecular-iris angle (TIA) (Fig. 2B). AOD is defined as the length of the line drawn from the point on the endothelial surface 500 μm or 750 μm (AOD 500, AOD 750) anterior to the scleral spur to the iris surface perpendicular to the corneal endothelial surface. TIA is defined as an angle formed by the apex at the iris recess and the arms passing through the point on the meshwork 500 μm or 750 μm (TIA 500, TIA 750) from the scleral spur and the point on the iris perpendicularly opposite. Because the anterior iris surface profile may confound the measurement of the AOD/TIA, angle recess area (ARA) and trabecular-iris space area (TISA) were subsequently introduced by Ishikawa and Radhakrishnan, respectively.^17,18^ ARA is defined as a triangular area bordered by the anterior iris surface, corneal endothelium and a line perpendicular to the corneal endothelium drawn from a point 500 μm/750 μm (ARA 500/ARA750) anterior to the scleral spur to the iris surface.^17^ The ARA is, theoretically, a better measurement parameter than the AOD because it takes into account the whole contour of the iris surface rather than measuring at a single point on the iris as is the case with the AOD. TISA is a new parameter proposed by Radhakishnan for quantitative measurement of the ACA. The defining boundaries for this trapezoidal area are as follows: anteriorly, the AOD 500 or AOD 750; posteriorly, a line drawn from the scleral spur perpendicular to the plane of the inner scleral wall to the opposing iris; superiorly, the inner corneoscleral wall; and inferiorly, the iris surface.^18^ This parameter represents the actual filtering area more accurately when compared with the ARA because the TISA excludes the nonfiltering region behind the scleral spur.

**Table 1:** **Table 1:** Summary of system features of the Visante OCT and SLOCT

		*Items*		*Visante OCT*		*SLOCT*	
Light		Laser source		SLD		SLD	
Source		Center wavelength		1310 nm		1310 nm	
		Spectral bandwidth		N/A		50 nm	
		Optical power		<6.5 mW		<0.2 mW	
Resolution		Axial resolution		~18 μm		~25 μm	
		Transverse resolution		~ 60 μm		20-100 μm	
		Pixel resolution (transverse × axial)		256~512 × 1024		215 in axial	
		Signal-to-noise ratio		N/A		>90 dB	
Scan speed		A scan frequency (Hz)		2048		200	
		A scan number/line		256		215	
		OCT frames/second		8		1	
Penetration		Opaque media		Yes		Yes	
		Limbus, sclera		Yes		Yes	
		Ciliary sulcus visible		No		No	
		Zonule visible		No		No	
Scan types		Maximum scan width × scan depth		16 × 8 mm		15 × 7 mm	
		Single scan		Yes		Yes	
		Dual and quadrant scans		Yes		No	
		Pachymetry map		Yes (8 meridians)		No	
Image		Eye contact		No		No	
Acquisition		Eye movement monitoring		Yes		Yes	
		Real-time image		Yes		Yes	
		Internal fixation target		Starburst pattern		No	
		Fixation angle adjustment		Yes		No	
		Accommodation adjustment range		– 35D to + 20D		No	

SLD: Superluminescent diode; N/A: Not available

Although UBM offers tremendous insight into the anterior chamber angle configuration and allows detailed imaging of the ciliary body and the posterior chamber, it is limited by being a close-contact immersion technique (Table 2). Inadvertent corneal indentation can cause artifacts, widening the angle measurement.^17^ With new ASOCT imaging techniques, detailed spatial relationships of the anterior segment structures can be visualized and objective ACA measurements can be performed in a non-contact manner. In addition, the use of infrared laser and real-time eye position monitor during examination permits the precise capture of angle morphology in the dark. With higher scan speed (8 frames/sec), SLOCT has the potential to provide valuable quantitative and spatial information regarding dynamic changes of the angle configuration which cannot be provided by standard gonioscopy and UBM.

The Reliability of ACA Measurement

Most of the important parameters for ACA quantitative measurement are based on the identification of the point of scleral spur. Reliable documentation of the angle dimensions is therefore dependent on precise and repeatable localization of the scleral spur. Sakata et al found that, on the same Visante OCT images, the intraobserver agreement in detecting the scleral spur (132 quadrants) was moderate to substantial with κ = 0.65.^19^ They also reported that, in the assessment of the exact scleral spur location, the distance between the scleral spur localized in the same image across 2 sessions was within 10 in 83% of the 78 quadrants assessed and within 20 in 90%. The location of the scleral spur on ASOCT images was less detectable in quadrants with a closed angle on gonioscopy (odds ratio = 0.54, p = 0.02) and also in images obtained in the superior and inferior compared with the nasal and temporal quadrants (64, 67, 75 and 80% respectively; p < 0.001).

The differences in ACA measurements in different lighting conditions have been investigated with UBM and ASOCT.^17,20,21^ Leung et al also described the dynamic ACA changes induced by dark-light changes by Visante OCT through real-time video recording.^22^ They found that the AOD and TISA decreased linearly with increasing pupil size in most cases (85.5% in AOD and 90.9% in TISA). It was estimated that for each mm change in pupil size, there was an average of 94 change in the AOD 500 and 0.035 mm^2^ change in the TISA. Although significant differences of angle measurements were found between light and dark conditions, good repeatability and reproducibility was achieved as long as the lighting condition had been standardized by ASOCT with the intersession ICC for AOD and TIA measurements being higher than 0.91.^20,23^ The intersession CVw for angle measurements by Visante OCT was less than that of UBM (Visante OCT: 6~11%, UBM: 16~18%).^20,24^

Significant correlations were found among ACA measurements by ASOCT, gonioscopy and UBM.^25,26^ In general, the correlation in detecting a closed ACA quadrant using ASOCT and gonioscopy was fair with a k of 0.4. But ASOCT tended to detect more closed angles than gonioscopy, particularly in the superior and inferior quadrants.^19^ There was no significant difference in angle measurements between ASOCT and UBM in either nasal or temporal quadrants, but a significant higher AOD measurement was observed by ASOCT in the superior and inferior angles compared with UBM.^8,25^ Of note, although SLOCT and Visante OCT generally had no significant difference in angle measurements, the two available ASOCT models had poor correlations in ACA measurement despite comparable pupil diameters obtained, with the spans of 95% LOA of the nasal/temporal angle measurements between them being 437 nm/531 nm, 0.174 mm^2^/0.186 mm^2^, and 25.3728.0° for AOD, TISA and TIA, respectively.^27^ The poor correlation is likely related to differences in the choice of refractive indexes in the calculation of anterior segment dimensions, algorithms for image dewarping, the exact scan locations and the state of accommodation.

Clinically, ASOCT has been applied to the observation of ACA change after glaucoma surgeries, such as laser peripheral iridotomy (LPI), argon laser peripheral iridoplasty (ALPI), trabeculectomy combined with cataract extraction and intraocular lens implantation, etc.

**Table 2:** **Table 2:** Comparisons of ASOCT with ultrasound microscopy (UBM)

		*ASOCT*		*UBM*	
Scan source		Infra-red light (1310 nm)		Ultrasound (50~80 MHz)	
No corneal indentation		Yes		No	
Examination position		Sitting		Supine	
Precise scanning		Yes		No	
Imaging under dark		Yes		No	
Internal fixation		Yes		No	
A scans/line		256		256	
Scan speed (Frame/sec.)		~ 8		~ 8	
Axial resolution (nm)		~ 18		~25 μm	
Transverse resolution (nm)		~ 60 μm		~50 μm	
Scan depth (mm)		~16.0×~8.0		5.0 × 5.0	
CVw for ACA measurement		6~11%		16~18%	
Cillary body visualization		No		Yes	

Anterior Chamber Depth (ACD) and Anterior Chamber Volume (ACV)

Shallow anterior chamber is regarded as a cardinal risk factor for primary angle closure and therefore, anterior chamber depth (ACD) (Fig. 2A) measurement has shown to be a promising screening parameter for angle closure.^28^ With both UBM and OCT, ACD is most often measured as the axial distance from the internal corneal surface to the lens surface.^29^ Using a prototype ASOCT, Baikoff observed that the ACD increased over the early years of life, peaking at around 15 to 20 years, then slowly decreasing until 80 years.^30^ Their results also showed that ACD reduced by a mean of 30 μm with 1.0 diopter of accommodation.

Anterior chamber volume (ACV) (Fig. 2A) is another useful parameter for detecting individuals at risk of developing primary angle closure.^31,32^ Upon examination, the ASOCT proved to be reliable in ACV measurement with CVw less than 0.96%.^18,33^ With the aid of Visante OCT, Lei et al observed that the ACV increased significantly from 73.86 to 84.14 μl after laser peripheral iridotomy (p < 0.001).^34^

Imaging of Filtering Blebs

**Fig. 3:**
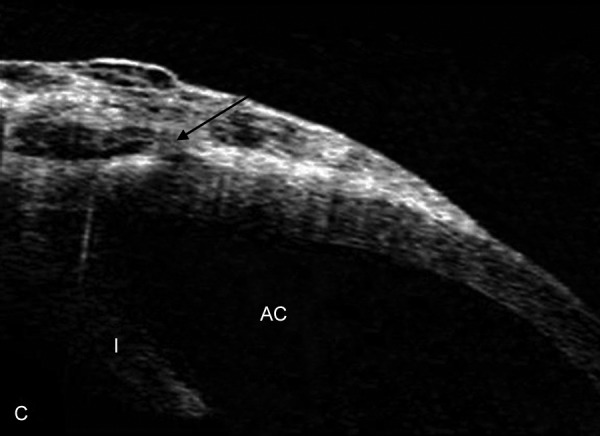
Filtering blebs: AS-OCT showing elevated functioning filtering blebs. The bleb is moderately elevated and homogeneously spongy with fluid-filled spaces

Blebs imaging after trabeculectomy was first reported with UBM.^35-37^ Other methods of bleb imaging that have been reported in the past include *in vivo* confocal microscopy^38^ and AS-OCT.^39,40^ The noncontact nature of OCT imaging provides a much safer approach to examine the intrableb morphology (Fig. 3) in the early postoperative period, thereby offering a unique opportunity to study the healing and remodeling process inside the blebs longitudinally.^38-42^ Using ASOCT, four-different patterns of intrableb morphology, including diffuse filtering blebs, cystic blebs, encapsulated blebs and flattened blebs were identified and found to be closely related to slit-lamp appearance and bleb function.^39^ Combining both clinical and bleb imaging information could provide a new perspective toward understanding the different surgical outcomes after trabeculectomy. More recently, Singh et al have reported bleb morphology in patients using spectral domain OCT (Cirrus HD-OCT, Carl Zeiss Meditec Inc., Dublin, CA, USA). In this study the SD-OCT could reveal details of the bleb wall but could not image the deeper structures such as internal ostium or flap position.^43^

Tominaga et al^44^ assessed the postoperative filtering bleb function using AS-OCT. In this study the height and extent of the internal cavity and the bleb height were not correlated with the IOP. The mean IOP in eyes with a low-reflectivity bleb wall was significantly (p = 0.001) lower than in eyes with a high-reflectivity wall. The bleb wall thickness was negatively (p = 0.004) correlated with the IOP.

Pfenninger et al^45^ analyzed the morphology of filtering blebs shortly after trabeculectomy surgery using AS-OCT. They found that the internal reflectivity of the fluid-filled cavity of the filtering bleb correlated very well to the IOP with R(2) = 0.3762, p < 0.0001. This study may have future impact in evaluating the postoperative success or failure of filtering blebs.

**Fig. 4:**
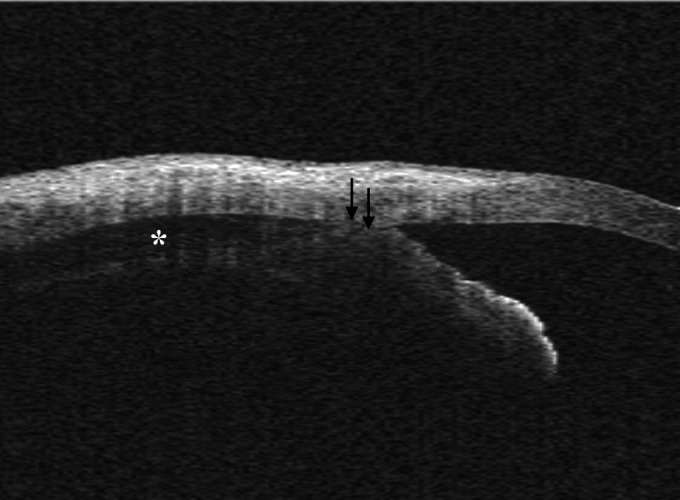
Cyclodialysis cleft: AS-OCT reveal the separation between the longitudinal muscle of the ciliary body and the scleral spur (arrows). Note the supraciliary effusion (asterisks)

*Other conditions cyclodialysis cleft:* In ocular trauma and in postoperative period, AS-OCT (Fig. 4) should be kept in mind for evaluating any trauma/postoperative-associated changes in ocular anatomy. Cyclodialysis is a separation of the longitudinal muscles of the ciliary body from the scleral spur and is often accompanied by a supraciliary effusion.

**Fig. 5:**
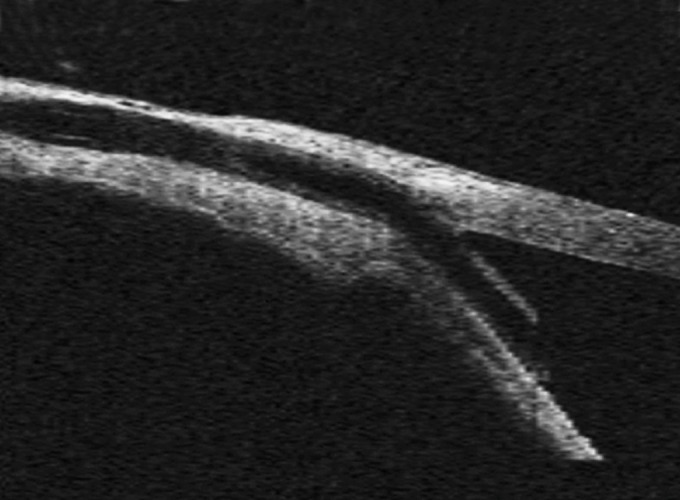
Glaucoma drainage implant: AS-OCT showing the path of the tube from the anterior chamber

*Glaucoma drainage implants:* The position, patency and course of drainage tubes can be ascertained using AS-OCT (Fig. 5) ASOCT would be especially helpful in assessing the tube inserted into the ciliary sulcus. The anatomic relationships can be assessed, as can compression of the tube at the scleral entry site.

## SUMMARY AND CONCLUSION

The major advantages of the newer devices are the non-contact nature of examination, high scan speed, good repeatability and reproducibility for quantitative and qualitative measurements and cross-sectional visualization of anterior segment structures. Since, ASOCT can visualize entire anterior chamber, all the essential parameters for detection of angle closure/narrow angle can be examined in a single scan. The ASOCT would become an essential tool for screening PAC, making screening programs for PACG more feasible and less doctor-dependent (Leung, Eye 2011). The application of ASOCT has led to a better understanding of anterior segment diseases. It can now be readily quantified making longitudinal follow-ups and assessments possible. The use of newer anterior segment imaging devices could well be the start of a new era for ophthalmic diagnosis. With the advent of Fourier domain OCT, the diagnostic capacity of ASOCT has increased many folds. Although still in its preliminary stages, it has the potential to transform ASOCT into a practical and decision-making tool.
